# Patient-reported evaluation of oral/dental health in oncology care—a need and opportunity to support medically necessary oral care needs

**DOI:** 10.1007/s00520-025-09316-w

**Published:** 2025-03-07

**Authors:** Joel B. Epstein, Ira R. Parker, Shalya Anand

**Affiliations:** 1 City of Hope National Medical Centre, Duarte, CA and Cedars-Sinai Medical System, Los Angeles, CA USA; 2 La Jolla, CA USA; 3 Erasmus School of Health Policy & Management, Erasmus University Rotterdam, Rotterdam, The Netherlands; 4 Center for Adaptive Rationality, Max Planck Institute for Human Development, Berlin, Germany

In the United States, it is estimated that there are 60,000 new head and neck cancer (HNC) patients annually and an estimated 500,000 HNC survivors [1]. At the time of HNC diagnosis, dental disease is estimated to be present in 40% of patients and approximately one third require dental extraction prior to cancer therapy [2]. Acute and chronic oral complications due to cancer therapies are clearly documented which affect the quality-of-life of patients with cancer [3]. Furthermore, timely oral health assessment, and medically necessary dental treatment in persons with cancer, has been associated with improved overall survival, and reduced morbidity and mortality [3, 4].

Assessment and management of oral and dental care across the trajectory of oncology care from diagnosis through survivorship are a well-recognized need. Guidelines have been developed to assess and manage oral/dental needs and oral complications before and during cancer care [4–21]. These guidelines for oral care are endorsed by several organizations and societies including the American Society of Clinical Oncology, the National Cancer Centre Network, the Multinational Association of Supportive Care in Oncology / International Society for Oral Oncology, the European Society of Medical Oncology, the American Society of Therapeutic Radiation Oncology, and the American Head and Neck Society and others.

Best practices and standards of oral care are essential components across the cancer continuum in head and neck cancers, some solid and liquid tumours, hematopoietic cell transplant and CAR-T therapy, specific medical interventions including use of bone modifying agents and anti-angiogenic agents, targeted cancer therapies, and immunotherapies. Generally, however, there is limited application of integrated oral and dental care for patients with head and neck cancers and across oncological care.

In the setting of head and neck cancers, and specifically in those patients planned to receive head and neck radiation therapy, the standard of care is *pretreatment dental assessment*. Acute dental needs and chronic dental and periodontal conditions that may require future surgical management within the radiated volume must be addressed prior to radiation therapy to reduce the risk of osteoradionecrosis. In addition, dental/maxillofacial reconstruction for support of surgically related conditions is essential. Hyposalivation/xerostomia that may follow therapy increases risk to oral and dental health and overall oropharyngeal function. This may result in heightened risk for rampant dental disease and the enhancement of pain, dysphagia, and malnutrition leading to poor quality of life [3].

In liquid tumours and transplantation care, chronic local oral/dental infection is of specific concern during periods of neutropenia. Patients with pancytopenia are at risk for significant local and systemic infection at a time when definitive intervention is not possible. In solid tumours in the head and neck cancer populations and in distant tumours of epithelial origin, including cancers of breast, colon, lung, and prostate, direct oral cytotoxicity may occur, resulting in acute or chronic oral complications such as mucositis, xerostomia, oral and pharyngeal pain, and a higher risk of infections [3]. Timely oral/dental evaluation and appropriate treatment in the context of overall patient management can improve the quality-of-life of patients with cancer.

In the oncology setting, high-risk patients including HNC and transplantation are recommended to obtain oral/dental evaluation and medically-necessary dental treatment prior to cancer care. Recently, the Centers for Medicare and Medicaid Services (CMS) has instituted a new reimbursement ruling, implemented in 2024, to support oral/dental evaluation and care prior to cancer therapy due to CMS’s acknowledgement that medically-necessary dental care needs impact oncology outcomes and patient quality-of-life [22, 23]. These needs have been recognized by CMS which initiated payment for dental services that are directly linked to other covered medical services prior to cancer therapies (including HNC, SCT, CAR-T, bone anti-resorptive treatment [22–25]. This initiative is a step towards addressing dental needs as it has been estimated that less than 50% of patients receive oral/dental care prior to cancer care [22–26]. Furthermore, in the oncology setting, integration of regular access to dental care may be influenced by factors such as limited interprofessional education/training, dental-medical workforce issues, oral health literacy factors, and patient financial toxicity [3]. In addition, dental providers may be poorly prepared to understand the complexity and impact of cancer therapy upon dental care, leading to both under- and over-treatment, due to limited multidisciplinary training as well as poor workforce accessibility and affordability [3].

## Patient-reported oral/dental health outcomes

Patient-reported oral/dental health outcomes tools (PRO) have the potential to provide relevant oral status information that may facilitate the addressing of oral care needs of cancer patients [25]. PRO tools have a key role to play in overall patient care and are increasingly recognized as critical elements in improving patient care [25]. The goal of the oral/dental PRO is to identify specifically oral/dental care needs, support oral/dental evaluation, and identify patients with urgent dental needs that require medically necessary management [25].

A PRO screening tool (Appendix:1) to predict oral/dental care needs based upon patient report has been shown to have the capacity to screen for oral conditions and diseases, and predict treatment needs potentially applicable across oncology settings, including specific cancers and/or being from certain age-demographic cohorts including paediatric, adult and elderly populations [25].

The PRO study cohort included 1,004 patients [25]. The patient-generated PRO was compared to an oral/dental evaluation by a “blinded” panel of periodontal specialists. The PROs identified 114 patients with urgent oral/dental care needs, as defined by specialist periodontist evaluation. The study assessed total scores in patient response and found diagnostic capability predicating the need for care.

The PRO survey included questions addressing PRO oral/dental symptoms and overall report of oral health. The study assessed the symptoms more frequently reported in the urgent care group compared to the non-urgent care patients: mouth sores (19.3% v 6.0%), altered speech or eating due to mouth problems (17.5% v 7.3%), loose teeth (23.7% v 9.8%), bleeding in the mouth (21.1% v 12.2%), broken teeth (33.3% v16.6%), all statistically significant in univariate analysis (*P* < 0.01). In multivariate analysis, swelling in the mouth or jaw (OR 4.09, 95% CI2.15–7.81), wisdom tooth problems (OR 2.45, 95% CI 1.61–5.17), pain with biting (OR 2.50, 95% CI 1.48–4.26), loose teeth (OR 2.32, 95%CI 1.24–4.35), and broken teeth (OR 1.72, 95%CI 1.10–2.93) were all statistically significant (*P* < 0.01). In evaluation of total score on the survey, a cutoff of 1.5 corresponded to a sensitivity of 0.84 and specificity 0.55.

The study emphasizes that the PRO oral/dental screening may be applicable for screening in patients with cancer and in identifying lower risk patients that may have significant dental needs in the oncology setting. The “lower risk patients” includes non-head and neck cancer solid tumours in which a subset may have oral/dental, oncology treatment related complications. In those patients identified by a PRO instrument, referrals for oral/dental examinations and determination of care needs can follow.

Potential impact of PRO for oral/dental health and identification of acute and chronic, medically necessary dental needs:

1. Facilitation of urgent referrals for high-risk patients.
2. Identifying patients with acute and significant oral conditions who are undergoing lower risk cancer therapy and triaging them for dental/oral care.
3. Identifying patients with high-risk oral conditions which may have a life-long impact with cancer treatments (e.g. head and neck RT patients).

This could promote timely care through appropriate screening, prevention, and maintenance for oral health by empowering caregivers, older patients with poor oral health and those with limited capacity to perform necessary oral hygiene.

Cancer-related medically necessary oral/dental care is recognized as an essential element of best practices cancer care, an important issue for cancer patients, practitioners, policy makers, and advocates. To address oral care needs in the oncology setting, community dental care providers require training from dental and oncology practitioners to enhance their competencies to provide appropriate referral and medically necessary oral care.

PRO tools may have the potential to promote heightened oral/dental care coordination with overall oncology care management across the entire cancer care continuum. Oral/dental PRO can empower patients, caregivers, and care-givers by identifying oral adverse events so that timely, appropriate care might be sought. This could promote and timely care through appropriate screening, prevention, and maintenance for oral health by empowering care-givers, older patients with poor oral health, and with limited capacity to perform necessary oral hygiene.

Considering the use of new and innovative cancer treatments and technologies, an enhanced and vigorous research agenda focusing upon adverse event-centred, oral/dental research, including standardized, validated, treatment-specific oral supportive care instruments (e.g. the PRO tool) needs to be both promoted and advocated for by both the oncology and oral/dental practitioner and research “communities”.

## Data Availability

No datasets were generated or analysed during the current study.
